# Anion-based electrolyte chemistry for sodium-ion batteries: fundamentals, advances and perspectives

**DOI:** 10.1039/d5sc08154h

**Published:** 2025-12-09

**Authors:** Shu-Yu Li, Yong-Li Heng, Zhen-Yi Gu, Xiao-Tong Wang, Yan Liu, Xin-Ru Zhang, Zhong-Hui Sun, Dai-Huo Liu, Bao Li, Xing-Long Wu

**Affiliations:** a Department of Chemistry, Northeast Normal University Changchun Jilin 130024 P. R. China xinglong@nenu.edu.cn; b State Key Laboratory of Integrated Optoelectronics, MOE Key Laboratory for UV Light-Emitting Materials and Technology, Northeast Normal University Changchun Jilin 130024 P. R. China xinglong@nenu.edu.cn guzy166@nenu.edu.cn cczhsun@gzhu.edu.cn; c Collaborative Innovation Center of Henan Province for Green Manufacturing of Fine Chemicals, School of Chemistry and Chemical Engineering, Henan Normal University Xinxiang Henan 453007 P. R. China

## Abstract

The scarcity of lithium resources and cost concerns have driven the development of sodium-ion batteries (SIBs), which utilize more abundant and lower-cost resources, as a promising alternative energy storage technology. As a key component of batteries, the electrolyte directly influences their energy density, rate capability, cycle life, and safety. However, conventional electrolytes face limitations such as poor high-voltage compatibility, inferior low-temperature performance, and unstable electrode–electrolyte interfaces, which severely hinder the commercialization of SIBs. In recent years, anion-regulated electrolytes have emerged as a research focus due to their unique anion-dominated solvation structures, demonstrating significant potential in enhancing interfacial stability, facilitating ion transport, improving high- and low-temperature performance, and strengthening safety. This review systematically elucidates the fundamental chemical characteristics and solvation structure design of anion-based electrolytes for SIBs, summarizes the influence of the mechanisms of different anion types on electrochemical performance, and explores the expanded application of anions in new systems. Finally, it outlines future directions and challenges in the field, providing valuable insights for the rational design of high-performance SIB electrolytes.

## Abstract 2

This review examines anion-regulated electrolytes for sodium-ion batteries, including solvation structure and mechanism to enhance interfacial stability, ion transport, and extreme-temperature performance, while also outlining future directions.

## Graphical abstract

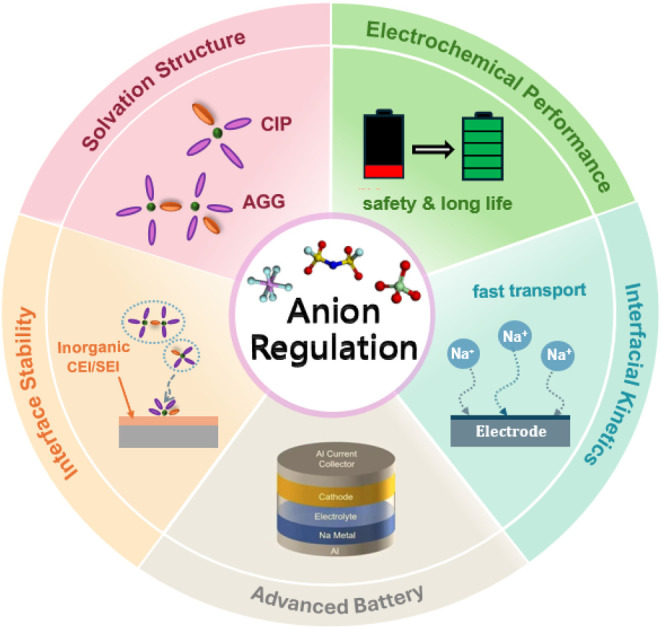

## 1 Introduction

The explosive growth of the new energy industry has increasingly highlighted the scarcity and supply–demand imbalance of lithium resources. Global lithium reserves are relatively limited and highly concentrated geographically, which not only drives up raw material costs but also poses potential constraints on large-scale applications, emerging as a critical bottleneck hindering the sustainable development of the industry.^1–5^ Under such circumstances, the search for novel energy storage systems that combine resource abundance with performance advantages has become an inevitable direction for industrial development. Driven by this demand, sodium-ion batteries (SIBs) have emerged as an ideal alternative. Compared to lithium, sodium resources are vastly more abundant and widely distributed in oceans, salt lakes, and underground rock formations, offering inherent advantages such as broad availability and ease of access. More importantly, SIBs share a highly similar working principle with lithium batteries—both operate based on a “rocking-chair” intercalation/deintercalation mechanism for charge storage and release. This fundamental similarity enables significant technological inheritance in areas such as electrode material design and battery architecture, laying a solid foundation for the rapid iteration and industrial adoption of sodium battery technology.^6–11^ It is noteworthy that the electrolyte, often regarded as the “blood” of a battery, serves as a central component in the battery system, fulfilling multiple critical functions including ion transport, interface formation, and safety assurance. Its physicochemical properties directly govern key performance metrics of the battery, such as energy density, fast-charging capability, cycling stability, and operational safety.^12–15^ Given this context, research on electrolytes for SIBs is of great significance for promoting the maturation and application of sodium battery technology.

**Fig. 1:**
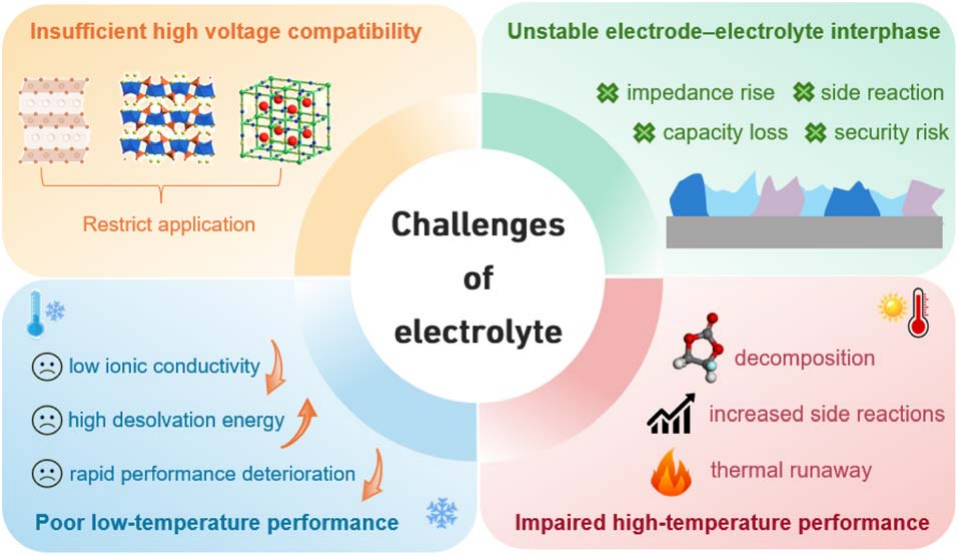
Challenges for SIB electrolytes.

Currently, the development of electrolytes for SIBs still faces several critical challenges that hinder their large-scale commercial application, as illustrated in Fig. 1. Firstly, insufficient high-voltage compatibility restricts the utilization of high-capacity cathode materials, thereby limiting further improvement in energy density and impairing long-term cycling stability.^16–18^ Secondly, the unstable electrode–electrolyte interphase (EEI) tends to trigger continuous side reactions, consuming active Na^+^ and reducing both coulombic efficiency (CE) and reversible capacity, while also compromising battery safety and cycle life.^19–21^ Moreover, the performance across a wide temperature range is poor. At low temperatures, slow ion transport and high desolvation barriers lead to a sharp performance decline,^22–25^ while at elevated temperatures, severe interfacial side reactions and facile electrolyte decomposition occur.^26,27^ Conventional electrolyte systems, limited by the intrinsic properties of their solvent–salt combinations, struggle to systematically address these issues. Therefore, designing novel electrolyte systems has become a crucial step in advancing SIB technology.

In recent years, anion-regulated electrolytes have garnered significant attention from both academia and industry as an emerging strategy. By facilitating an anion-dominated solvation structure, these electrolytes effectively modulate ion transport and interfacial chemistry, directly addressing several bottlenecks in current battery technologies. Specifically, anion-regulated electrolytes demonstrate notable advantages in the following aspects: enhancing EEI stability and suppressing side reactions;^28,29^ facilitating ion transport and accelerating desolvation, thereby improving low-temperature and fast-charging performance;^30,31^ increasing oxidation resistance to widen the electrochemical stability window, enabling compatibility with high-voltage cathodes;^32–34^ and contributing to the formation of a more robust solid electrolyte interphase (SEI), which enhances the overall safety of the battery.^35,36^ Furthermore, this kind of electrolyte provides the possibility for the development of new battery systems (*e.g.*, dual-ion batteries and anode-free batteries), expanding the application scenarios of SIBs.^37,38^

Against this background, this paper systematically reviews the research status and development trends of anion-regulated electrolytes. It elucidates their basic structure and solvation chemistry, summarizes key recent research advances in SIBs, and discusses the design principles for different anion types and their coordination environments. This work aims to provide a theoretical foundation and a materials perspective for understanding and designing high-performance anion electrolytes. Finally, the article also outlines future research directions and technical challenges in this field, with the goal of informing the design and application of electrolytes for SIBs.

## 2 Characteristics of anion solvation structures

Anion-based electrolytes, as an emerging electrolyte system, have become one of the key research directions in the field of SIBs in recent years. By strategically modulating the structure and chemical properties of anions, such electrolytes are expected to address key bottlenecks in the performance and interfacial behavior of conventional SIBs. Unlike traditional solvation structures that rely primarily on “cation–solvent” interactions, anion-dominated solvation structures emphasize the regulation of anion–solvent–cation interactions, thereby constructing an electrolyte environment better aligned with functional requirements.^39^ In such structures, anions extensively enter the first solvation shell of sodium ions, forming contact ion pairs (CIPs) or even aggregates (AGGs).^38,39^ In contrast to the solvent-separated ion pair (SSIP) configuration, where anions are largely excluded, the aggregated configurations (CIPs and AGGs) effectively suppress the decomposition of solvent molecules and facilitate the formation of an anion-derived SEI or cathode electrolyte interphase (CEI). These interphases typically exhibit higher stability, compactness, and abundant inorganic components, thereby significantly enhancing electrode interfacial stability and ion transport efficiency.^40–42^ Moreover, conventional cation-dominated solvation structures are governed by strong cation–dipole interactions, which tend to increase the desolvation energy barrier of Na^+^ at low temperatures, resulting in sluggish kinetics and promoting the formation of organic-rich, less stable SEI/CEI layers—factors that severely restrict the low-temperature performance of SIBs.^43,44^ In contrast, anion-involved solvation structures significantly reduce the desolvation energy barrier and improve the interfacial reaction kinetics. They promote the formation of thinner, more uniform, and inorganic-rich SEI/CEI layers. As a result, such structures enable efficient Na^+^ transport and stable cycling over a wide temperature range, particularly under low-temperature conditions.^45–47^

**Fig. 2:**
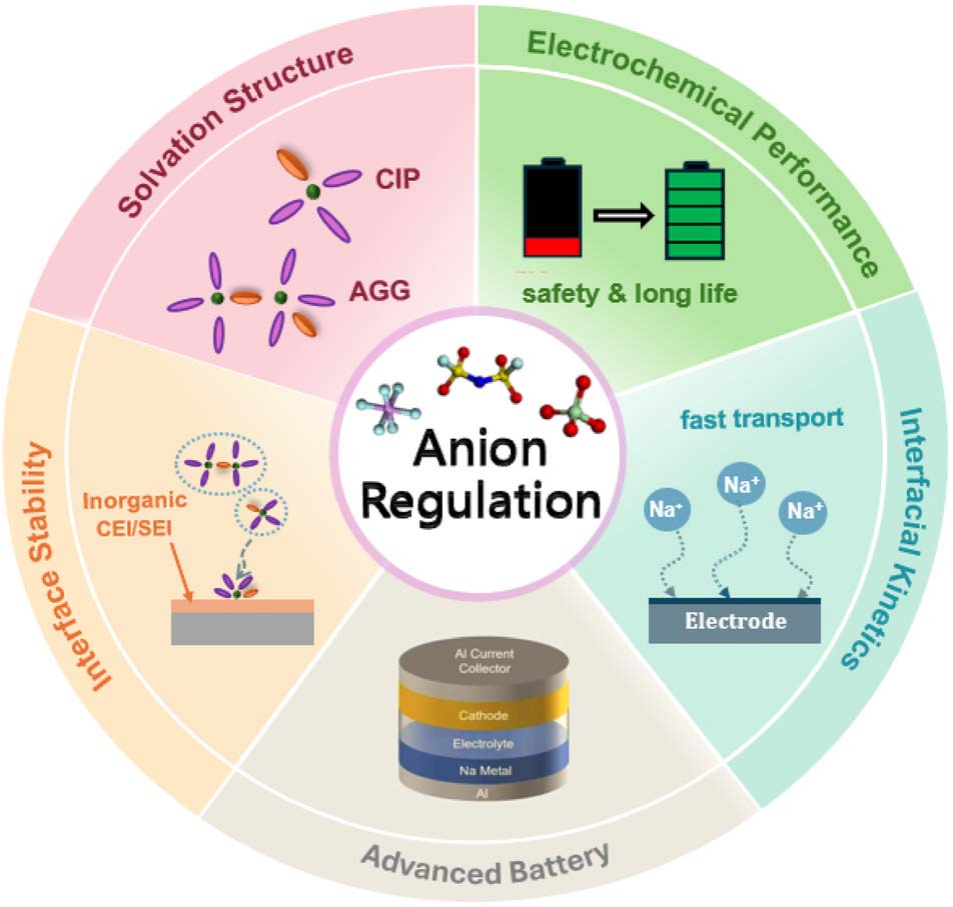
Schematic diagram of the anion regulation effect.

Hence, the anion plays a crucial regulatory role in several key aspects, including the optimization of the solvation structure, enhancement of electrochemical performance, improvement of interfacial stability and kinetics, and expansion of applications for novel batteries (schematic diagram shown in Fig. 2). The role of anions in solvation structures has evolved from that of a passive charge carrier to a critical regulating factor—a paradigm shift from a “supporting role” to a “leading role”. This transformation provides a novel design strategy for developing next-generation high-performance sodium battery electrolytes.

## 3 Involvement of solute anions in the solvation structure

### 3.1 Single anion

#### 3.1.1 NaPF_6_-based electrolyte

**Fig. 3:**
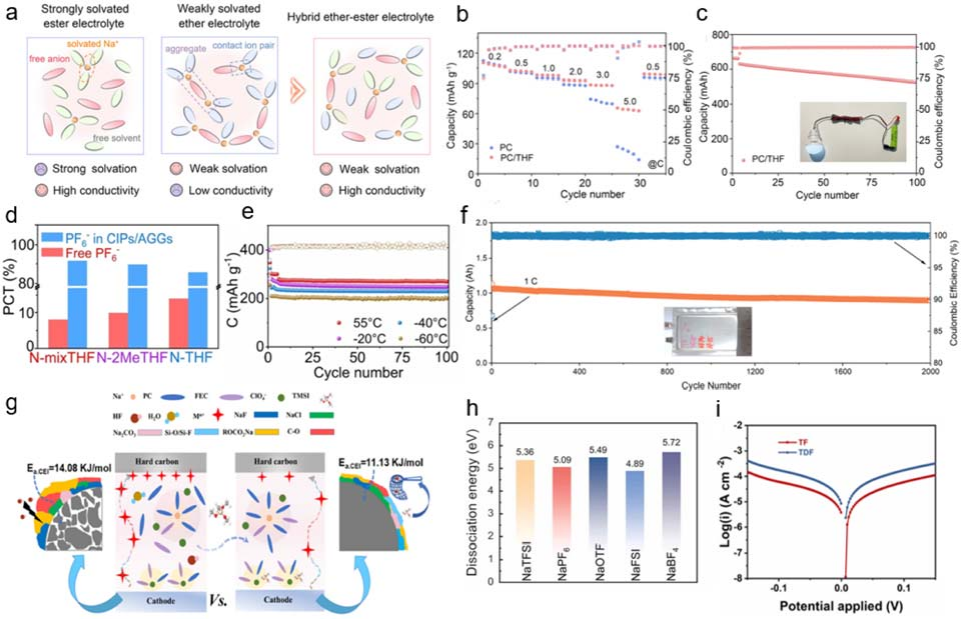
(a) Schematics of solvation structures and their merits of the three electrolytes. (b) Rate capability of a PB‖HC cell. (c) Long-term cycling performance of a PB‖HC cylindrical cell.^50^ Copyright 2024 Wiley-VCH GmbH. (d) Ratio of different coordinated structures. (e) Long cycling performance in N-mixTHF at different temperatures (100 mA g^−1^ beyond −20 °C, 50 mA g^−1^ at −40 °C, and −60 °C).^43^ Copyright 2024 Wiley-VCH GmbH. (f) Cycling performance of the HC‖NFPP pouch cell at 1C.^53^ Copyright 2024 American Chemical Society. (g) Anion-induced uniform and robust CEI for layered metal oxide cathodes of SIBs.^54^ Copyright 2024 American Chemical Society. (h) Dissociation energy of various Na salts. (i) Tafel curves of TDF and TDF electrolyte. Copyright 2025 Elsevier.

NaPF_6_ is the most widely used sodium salt and currently represents the dominant choice for electrolytes in SIBs. The PF_6_^−^ anion exhibits weak interaction with Na^+^, which promotes Na^+^ desolvation and enhances the charge transfer kinetics.^48^ Introducing weak coordination solvents is a common method to construct anion-enhanced solvated structures. For example, Qiu *et al.* introduced a weakly-coordinating tetrahydrofuran (THF) solvent, which promoted the participation of more PF_6_^−^ anions in the inner Na^+^ solvation sheath, increasing the coordination number from 0.49 to 1.84.^49^ Compared to conventional carbonate-based electrolytes, the designed hybrid electrolyte is expected to exhibit a weakly solvating, anion-enhanced structure and high ionic conductivity (Fig. 3a). The PC/THF system features an anion-enhanced solvation configuration, which facilitates the desolvation process and promotes the formation of an anion-derived inorganic-rich interphase layer. Combined with the entropy effect of ester–ether mixing that contributes to enhanced ionic conductivity, the Prussian blue‖hard carbon (PB‖HC) full cell shows significantly improved rate performance (Fig. 3b). Moreover, an industrial-grade PB‖HC 18650 cylindrical cell achieves a capacity retention of 82.5% after 100 cycles (Fig. 3c). Similarly, Li *et al.* designed a weakly solvated electrolyte with weakened Na^+^-dipole interaction. The reduced Na^+^-solvent binding strength is beneficial for more anions to participate in the solvation sheath, retaining the anion-enhanced solvation structure from room temperature down to low temperatures.^43^ According to the Raman spectroscopy results, the ratio of free/coordinated PF_6_^−^ in different electrolytes is shown in Fig. 3d; the target electrolyte N-mixTHF exhibits the highest ratio of CIPs to AGGs, indicating greater participation of PF_6_^−^ in the Na^+^ solvation sheath and weakened ion–dipole interactions between Na^+^ and solvent molecules. This anion-enhanced solvation structure facilitates Na^+^ desolvation kinetics and promotes the preferential decomposition of anions at the anode, leading to the formation of an inorganic-rich SEI. This SEI effectively reduces the Na^+^ diffusion barrier and suppresses continuous electrolyte decomposition. As a result, the hard carbon anode delivers high reversible capacity and outstanding cycling stability even under extreme low-temperature conditions (Fig. 3e).

#### 3.1.2 NaClO_4_-based electrolyte

NaClO_4_ is considered as one of the preferred electrolyte salts for SIBs, owing to its advantages including fast ion migration, excellent chemical stability, high thermal stability, and low cost.^51,52^ Chen *et al.* reported an anion-dominated ion–solvent coordination structure in a conventional low-concentration electrolyte (1.22 M).^52^ The significantly enhanced Na^+^–anion interaction enables anions to dominate the Na^+^ coordination competition. This strengthened cation–anion interaction improves the electrochemical compatibility of the solvent. Consequently, an Ah-level HC‖Na_4_Fe_3_(PO_4_)_2_(P_2_O_7_) pouch cell exhibits an average CE exceeding 99.9% and a capacity retention of 84.5% over 2000 cycles (Fig. 3f), along with successful operation over a wide temperature range (−20 to 60 °C). Sun *et al.* modified the anion solvation structure by introducing a silane-based additive, which enabled the construction of an anion-derived CEI on the NaNi_1/3_Fe_1/3_Mn_1/3_O_2_ (NFM) cathode.^53^ Due to its stronger interaction with trimethoxymethylsilane (TMSI), compared to PC/FEC, the ClO_4_^−^–TMSI complex aggregates on the NFM surface, where it is oxidized and participates in forming the CEI. The activation energy for Na^+^ migration through the CEI decreased from 14.08 kJ mol^−1^ to 11.13 kJ mol^−1^, indicating that the TMSI-induced CEI is more stable and uniform, which facilitates Na^+^ diffusion kinetics and effectively preserves the structural integrity of the NFM cathode (the proposed mechanism is illustrated in Fig. 3g). The system demonstrates remarkable rate capability, cycling stability, and coulombic efficiency under harsh conditions, including elevated temperature (55 °C) and a high cut-off voltage range (2.0–4.3 V *vs.* Na^+^/Na).

Furthermore, Li *et al.* employed methyl butyrate, a weakly coordinating carboxylate co-solvent, to modulate ion–dipole interactions within a fluorine-free ester-based electrolyte, thereby promoting an anion-enhanced solvation structure.^54^ With more ClO_4_^−^ occupying the Na^+^ solvation sheath, the Na^+^ desolvation energy is significantly reduced, accelerating interfacial kinetics. Furthermore, robust anion-rich inorganic EEIs form on both the cathode and anode, effectively suppressing EEI dissolution and enhancing interfacial stability. A PB‖HC full cell achieved stable operation over a wide temperature range from −20 to 100 °C. This study demonstrates the potential of fluorine-free ester-based electrolytes with anion-enhanced solvation chemistry for advanced wide-temperature SIBs.

#### 3.1.3 NaBF_4_-based electrolyte

**Fig. 4:**
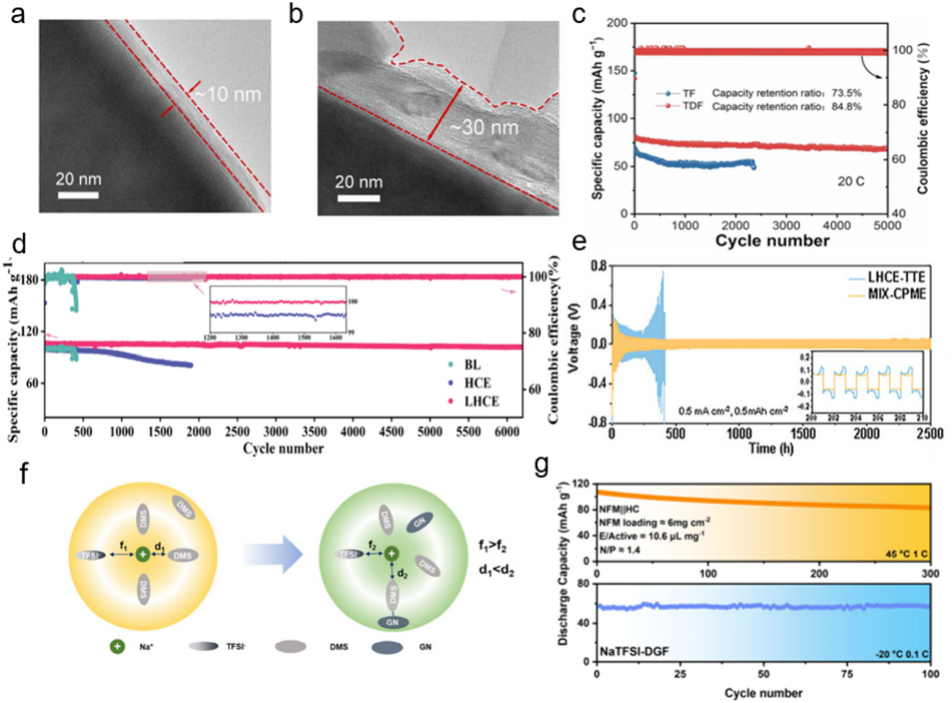
HRTEM images of NVPF cathodes after 100 cycles (a) in TDF electrolyte and (b) TF electrolyte. (c) Cycling performance of the Na‖NVPF coin cell using TF and TDF electrolytes cycled at 20C.^56^ Copyright 2025 Elsevier. (d) Cycling performance of Na‖NVP batteries at 1C.^58^ Copyright 2025 American Chemical Society. (e) Na‖Na symmetric cells with LHCE-TTE and MIX-CPME at a current density of 0.5 mA cm^−2^ and an areal capacity of 0.5 mA h cm^−2^.^59^ Copyright 2025 Wiley-VCH GmbH. (f) Schematic diagram of representative Na^+^ solvation structures in NaTFSI-DMS and NaTFSI-DG, respectively. (g) The cycling performance of NFM‖HC full cells at 45 °C and −20 °C.^62^ Copyright 2025 American Chemical Society.

NaBF_4_ is a functional electrolyte salt and a high-performance additive in SIB electrolyte, which has high electrochemical stability.^55^ Chen *et al.* used NaBF_4_ salt to trigger the weak solvation effect.^56^ By employing density functional theory (DFT), they calculated the dissociation energies of various sodium salts. A higher dissociation energy indicates greater stability of the substance. As shown in Fig. 3h, NaBF_4_ exhibits the highest dissociation energy (5.72 eV) among the studied salts, suggesting the strongest electrostatic attraction between Na^+^ and BF_4_^−^. This implies that BF_4_^−^ tends to enter the first solvation sheath, facilitating the formation of a weakly coordinated electrolyte. Concurrently, diethylene glycol dimethyl ether was introduced as a co-solvent to weaken the ion–dipole interaction between solvent molecules and Na^+^. Cyclic voltammetry tests indicate that in the optimized electrolyte, sodium symmetric cells (Na‖Na) achieve more reversible sodium metal deposition/dissolution behavior, which is attributed to the significantly enhanced interfacial reaction kinetics reflected by their higher exchange current density (Fig. 3i). Transmission electron microscopy (TEM) characterization of the cycled cathode showed a thinner and more uniform CEI, signifying fewer side reactions and improved interfacial stability (Fig. 4a and b). Consequently, the constructed anion-enhanced solvation structure promotes rapid Na^+^ transport and desolvation, leading to the formation of a robust, inorganic-rich electrode–electrolyte interphase. The Na‖Na_3_V_2_(PO_4_)_2_F_3_ (NVPF) pouch cell employing the optimized electrolyte exhibits a capacity retention of over 86.4% after 280 cycles at a charging voltage of 4.5 V. Meanwhile, the NVPF coin cell demonstrates stable cycling for 5000 cycles at a high current density of 20C (Fig. 4c). This approach not only widens the voltage window of sodium metal batteries (SMBs) but also ensures long-term stability and durability, offering new insights for developing high-energy-density batteries.

#### 3.1.4 NaFSI-based electrolyte

Sodium bis(fluorosulfonyl)imide (NaFSI), characterized by its unique [(FSO_2_)_2_N]^−^ anion structure, is renowned for its superior physicochemical and electrochemical properties, making it a promising candidate for high-performance SIBs.^57^ Zhou *et al.* introduced hydrofluoroether as a diluent in a high concentration electrolyte (HCE), forming a localized high-concentration electrolyte (LHCE), which effectively modified the solvation structure to enhance ion-pair coordination. This approach preserved the inherent CIPs and AGGs within the HCE, while strengthening the interaction between Na^+^ and FSI^−^ anions.^58^ Under the synergistic effect of diluent decomposition, an inorganic anion-rich SEI is easily generated on the surface of metal Na, which provides effective protection for the Na metal anode. The Na‖Na_3_V_2_(PO_4_)_3_ (NVP) cell employing this LHCE demonstrated significantly improved cycling performance, retaining 95.4% of its capacity after 6200 cycles (approximately 517 days), with an average CE exceeding 99.9%. This performance markedly surpasses that of cells using a baseline electrolyte (99.29%) and conventional HCE (99.67%) (Fig. 4d). Furthermore, the FSI^−^ anion-dominated solvation structure has been successfully extended to sodium–sulfur batteries. Manthiram *et al.* tailored the solvation structure to be anion-rich, thereby developing a resilient, inorganic-rich SEI that facilitates superior Na^+^ transport.^59^ Within this electrolyte environment, the Na‖Na symmetric cell achieves a cycling lifetime exceeding 2500 hours with excellent stability and a low overpotential (Fig. 4e), which benefits from the formation of the inorganic-rich SEI on the Na metal anode.

#### 3.1.5 NaTFSI-based electrolyte

Sodium bis(trifluoromethanesulfonyl)imide (NaTFSI) is also a sodium salt with excellent comprehensive properties, exhibiting better thermal stability and chemical stability and wider electrochemical window.^60,61^ Gao *et al.* leveraged the synergistic effects between solvents to modulate the Na^+^ solvation structure. This approach promoted the participation of more TFSI^−^ anions in the primary solvation sheath. The resulting anion-rich and loosely solvated structure concurrently enables faster Na^+^ transport/desolvation kinetics and the formation of a robust EEI.^62^ Specifically, there is hydrogen bond interaction between dimethyl sulfite (DMS) and glutaronitrile (GN). As shown in Fig. 4f, DMS solvent is considered as “pulling out” the solvation structure (d_1_ < d_2_), which induces the formation of the DMS and GN complex at the edge of the Na^+^ solvation structure. At the same time, this configuration exposes more coordination sites, promoting the participation of TFSI^−^ anions in the inner solvation structure (f_1_ > f_2_). As a result, the wide-temperature performance of the NFM‖HC full cells improved from −40 °C to 45 °C (Fig. 4g). Moreover, even at an ultralow temperature of −55 °C, the NFM‖Na half cells still exhibited reversible charge–discharge behavior. Combined with the broad operating temperature range and high mass loading of active materials, these features make such cells promising candidates for all-climate practical SIBs. In addition, Luo *et al.* effectively weakened the solvation energy of a carbonate-based electrolyte through the regulation of low solvation molecules in SMBs, reduced the proportion of free solvents, and realized the solvation sheath of primary Na^+^ ions enriched by TFSI^−^ anions.^60^ More FEC/TFSI^−^ participated in the formation of the interface; along with a reduction in free solvents, this led to the formation of a compact interphase enriched with inorganic NaF and salt-derived components. This robust layer effectively suppresses continuous SEI dissolution at the anode and parasitic side reactions at the cathode, thereby enhancing stability at both room temperature and elevated temperatures.

#### 3.1.6 NaOTF-based electrolyte

**Fig. 5:**
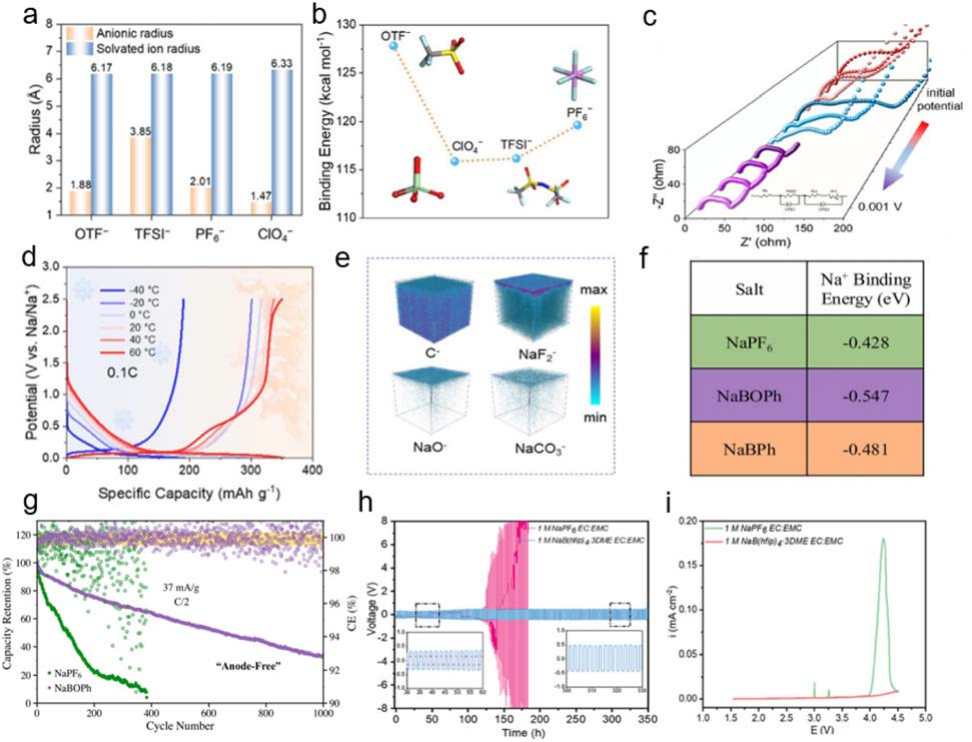
(a) Radius comparison of anion and solvated Na^+^ ions. (b) Binding energy of different anions. (c) *In situ* EIS at different potentials based on the optimized electrolyte. (d) Charge–discharge curves the of HC‖Na cell from −40 to 60 °C. (e) TOF^−^ SIMS 3D images of the OTF^−^-derived SEI film.^64^ Copyright 2025 Wiley-VCH GmbH. (f) Na^+^ binding energy tabulation of Na salts. (g) Cycling data of Na-LTO‖C–Al foil configuration.^65^ Copyright 2025 Elsevier. (h) Voltage *versus* time profile of the Na‖Na symmetric cell at a current rate of 0.5 mA cm^−2^ (1 mA h cm^−2^) (inset showing the zoomed potential profiles from 30 to 60 h and 300 to 330 h range). (i) Linear sweep voltammetry curves (scan rate – 0.1 mV s^−1^ up to 4.5 V).^66^ Copyright 2024 Wiley-VCH GmbH.

Sodium trifluoromethanesulfonate (NaOTF) is another common high-performance sodium salt. The SO_2_CF_3_^−^ anion, due to its delocalized electron structure, preferentially occupies the inner sphere of the Na^+^ solvation sheath, displacing conventional salt anions and solvent molecules, which is beneficial for the construction of a highly dynamic solvation microstructure.^63^ Xu *et al.* proposed an anion anchoring and interphase modulation strategy to tailor the microstructure of ether-dominated electrolytes, offering promising insights for the development of all-climate sodium storage based on hard carbon anodes.^64^ Through high-throughput theoretical calculations and screening strategies, OTF^−^ was identified as the optimal candidate due to its high binding energy with Na^+^ and minimal solvated ion radius (Fig. 5a and b), which facilitate the formation of an anion-dominated solvation sheath and enable rapid Na^+^ migration. *In situ* electrochemical impedance spectroscopy (EIS) was employed to monitor the dynamic evolution of impedance in the HC‖Na cell at different potentials. As shown in Fig. 5c, when discharged to 0.4 V, the impedance spectrum is dominated by a high-frequency semicircle, corresponding to a surface-adsorption sodium storage mechanism. Upon further discharge to 0.1 V, the impedance response evolves into three distinct semicircles, attributed to (i) Na^+^ desolvation at the electrode/electrolyte interface, (ii) ion transport through the SEI layer, and (iii) Na^+^ migration within the HC phase. Throughout this process, the total impedance gradually decreases, indicating enhanced Na^+^ transport kinetics. When the potential is further reduced to 0.001 V, the impedance spectrum transitions into two semicircles, suggesting the activation of a pore-filling sodium storage mechanism in the HC anode. The wide-temperature performance (−40 to 60 °C) depicted in Fig. 5d further demonstrates the practical potential of the anion-anchored ether-based electrolyte design for HC sodium storage. Moreover, as illustrated in Fig. 5e, the OTF^−^-induced inorganic-rich SEI, composed of NaF and Na_2_O, significantly promotes Na^+^ transport both in the bulk phase and across interfaces, while simultaneously enhancing long-term cycling stability.

#### 3.1.7 Other novel sodium salts

In addition to the solvation structure constructed by anions in classical sodium salts, a variety of novel sodium salts have recently been investigated to unlock further development potential for SIBs, address performance limitations, and promote anion-based electrolytes for sodium batteries.

Ban *et al.* designed a low-cost, fluorine-free sodium salt (sodium tetraphenoxyborate, chemical formula NaBO_4_C_24_H_20_, abbreviated as NaBOPh). This fluorine-free borate-based electrolyte has enabled breakthroughs in low-cost, high-performance sodium batteries, offering a new direction for sustainable energy storage technologies.^65^ By calculating the salt pairs and ion free energies, Na^+^ ions exhibit the strongest binding energy with this salt (Fig. 5f), indicating that the NaBOPh–diethylene glycol dimethyl ether (DG) electrolyte exhibits stronger Na^+^–anion pairing compared to NaPF_6_–DG or NaBPh–DG electrolytes, resulting in weakened Na^+^–DG interactions. Furthermore, the tighter binding geometry of cations in NaBOPh restricts the number of DG molecules available for coordination with Na^+^ ions, while the lack of fluorine in this salt structure contributes to more robust Na^+^–anion pairs. The distinctive molecular structure of NaBOPh leads to increased anion participation in the solvation sheath and reduced solvent coordination, thereby inducing the anion-rich interfacial chemistry observed in the borate-dominated SEI. These effects promote smoother and more uniform Na electrodeposition morphologies. The fluorine-free interphase demonstrates improved cycling performance across multiple battery configurations, including SIBs, SMBs, and anode-free sodium metal batteries (Fig. 5g).

Mitra *et al.* proposed a new electrolyte system based on a highly fluorinated borate anion ([B(hfip)_4_]^−^), consisting of 1 M Na[B(hfip)_4_]·3DME/EC:EMC. This design demonstrates remarkable performance enhancement in high-voltage SMBs compared to conventional NaPF_6_-based electrolytes.^66^ The Na[B(hfip)_4_]·3DME electrolyte system exhibited a consistently flat voltage profile for over 350 hours. In contrast, the NaPF_6_-based electrolyte showed a fluctuating voltage profile, with sodium stripping/plating lasting only 110 hours before a sudden voltage increase due to cell short-circuit (Fig. 5h). This suggests the formation of smooth, uniform, and non-porous Na metal deposition in the designed electrolyte system. Linear sweep voltammetry was conducted for Na‖Al cells to evaluate anodic stability. The 1 M NaPF_6_ EC:EMC electrolyte exhibited a sharp current increase beyond 4.0 V, indicating poor anodic stability of the [PF_6_]^−^-based system. In contrast, the [B(hfip)_4_]^−^-based system remained highly stable up to 4.5 V, with no significant current surge even after 4.0 V (Fig. 5i), demonstrating effective passivation of the Al current collector and suppression of further electrolyte oxidation.

**Fig. 6:**
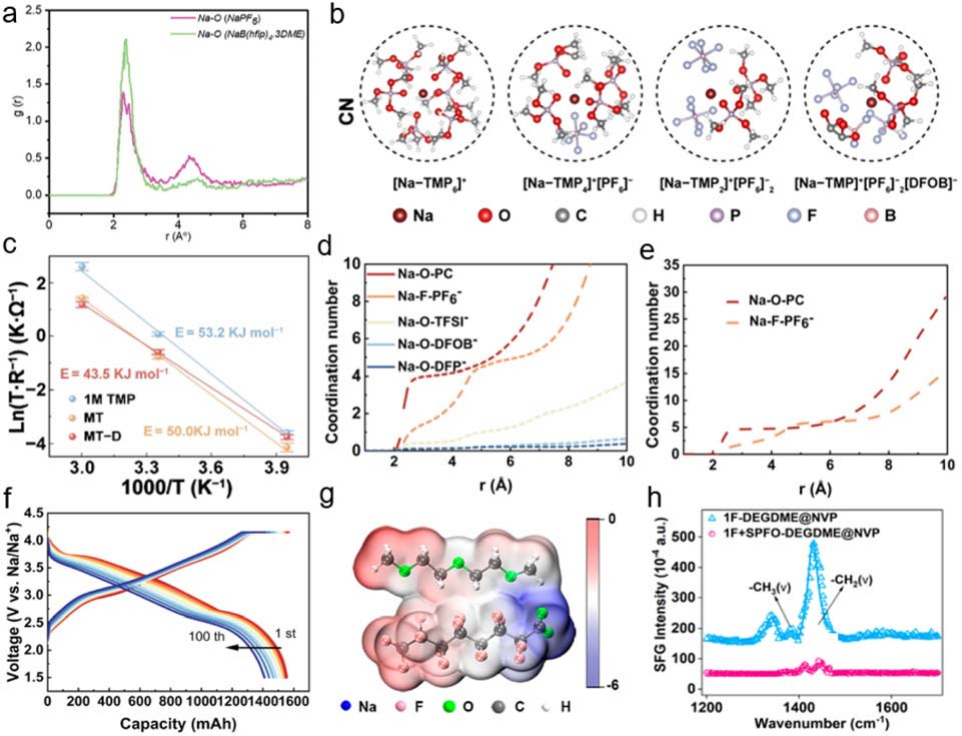
(a) Radial distribution functions of Na–O in Na[PF_6_] and Na[B(hfip)_4_]·3DME electrolytes.^66^ Copyright 2024 Wiley-VCH GmbH. (b) The representative solvation structures. (c) The fitted Arrhenius plots of 1 M TMP, MT and MTD, and the error bars represent the standard deviation of triplicates.^73^ Copyright 2024 Wiley-VCH GmbH. (d and e) CN of MUL electrolyte and STD electrolyte. (f) GCD profiles over multiple cycles in the voltage range of 1.5–4.15 V.^69^ Copyright 2025 Elsevier. (g) ESP simulation of SPFO–solvent complexes. (h) SFG signals of DEGDME between NVP and electrolytes.^93^ Copyright 2024 American Chemical Society.

To assess practical feasibility, the electrolyte was paired with sodium full-cell configurations. Excellent cycling stability and reversible capacity were achieved with high-voltage cathodes (NVPF@C@CNT and NFMO), along with significantly reduced transition metal dissolution. Analysis of the radial distribution function (RDF) in the *ab initio* molecular dynamics trajectories for Na–O in NaPF_6_ and Na[B(hfip)_4_]·3DME (Fig. 6a) reveals highly similar peak positions at 2.29 Å and 2.39 Å, respectively, representing the Na^+^–solvent distances within the primary solvation shell. A more pronounced difference appeared in the second solvation shell, with Na–O RDF peaks at 4.39 Å for NaPF_6_ and 4.57 Å for Na[B(hfip)_4_]·3DME, suggesting a less dense secondary solvation structure in the latter. This implies that Na^+^ may undergo easier desolvation and diffuse more readily to the electrode surface in this fluorinated borate-based carbonate electrolyte system. This study highlights the potential of weakly coordinating anions with similar properties for advanced electrolyte design.

These novel sodium salts are often designed by modifying the existing anions, with the core advantage of meeting the requirements of specific systems through tailored anions. For instance, introducing conjugated groups like benzene rings may alter the charge distribution of anions and affect frontier orbital energy levels, potentially conferring specific interfacial functions. However, the incorporation of bulky groups might limit solubility and ionic conductivity in conventional solvents, while also complicating synthesis. Consequently, these salts can be utilized to form stable interfacial films through their distinctive products or as sacrificial agents to improve electrolyte tolerance under extreme conditions.

### 3.2 Synergy of multi-anion systems

Based on our previous discussion of solvation structures governed by single anions, we now extend the scope to multi-anion systems. Compared with single-anion systems, multi-anion cooperation enables a more stable solvation structure by modulating the coordination behavior of different anions^67,68^ and introducing competitive coordination between them.^63,69^ Multi-salt formulations inherently facilitate the formation of heterogeneous interphases, enhancing structural and chemical stability, and thereby improving overall battery performance.^70–72^ The multi-anion strategy better reflects the concept of “proactively designed” solvation structures.

Wang *et al.* designed a unique dual-anion-aggregated Na^+^ solvation structure by employing cost-effective NaPF_6_ and NaDFOB(sodium difluoro(oxalato)borate) salts dissolved in trimethyl phosphate (TMP) and 1,1,2,2-tetrafluoroethyl-2,2,3,3-tetrafluoropropyl ether (TTE).^73^ This unique aggregate preferentially decomposes on the cathode, constructing a gradient CEI rich in phosphides and borides. Based on the trajectory data obtained from molecular dynamics (MD) simulations, the predominant solvation structures in the electrolyte were directly extracted (Fig. 6b). Among these, [Na–TMP_6_]^+^, [Na–TMP_4_]^+^[PF_6_]^−^, and [Na–TMP_2_]^+^[PF_6_]_2_^−^ represent typical solvation configurations corresponding to the SSIP, CIP, and AGG, respectively. The simulation results reveal the formation of a highly aggregated dual-anion structure, [Na–TMP]^+^[PF_6_]_2_^−^[DFOB]^−^, in the target electrolyte (MT–D), demonstrating the participation of DFOB^−^ in the solvation sheath and its coordination with Na^+^. Variations in Na^+^ solvation were further corroborated by the kinetic behavior across different electrolyte systems. The activation energy (*E*_a_) for (de)solvation, calculated using the Arrhenius equation (Fig. 6c), indicates that the MT–D electrolyte effectively reduces the energy barrier for the (de)solvation process, thereby facilitating rapid Na^+^ transport at the interface. This phenomenon is attributed to the anion-rich solvation structure formed in MT–D, which promotes the incorporation of anions into the solvation sheath. The involvement of anions reduces the energy required for complete dissociation of sodium salts, leading to a lower binding energy within the solvation structure. This accelerates desolvation kinetics and, in turn, enables rapid decomposition during cycling.

Zhang *et al.* proposed a simple strategy of anion competitive coordination for designing weakly solvating electrolytes. By introducing complex salts with high Na^+^-anion binding energy, abundant anions are retained within the solvation sheath, thereby optimizing both the Na^+^ solvation structure and the CE.^69^ In order to construct a more stable inorganic CEI, the anion competitive coordination weakly solvated electrolyte (MUL) containing 0.8 M NaPF_6_, 0.1 M NaTFSI, 0.05 M NaDFOB and 0.05 M NaDFP (sodium difluorophosphate) was prepared by adding composite salts into PC. For comparison, 1 M NaPF_6_ solution in PC was also prepared and analyzed, which was designated as STD. The selection of these salts was guided by their calculated Na^+^–anion binding energies, which follow the trend: NaDFP > NaDFOB > NaTFSI > NaPF_6_. Classical MD simulations were employed to investigate differences in the solvation structures between the STD and MUL electrolytes. Compared with STD electrolyte, the MUL system exhibits stronger coordination between anions and Na^+^, accompanied by a reduced coordination number of Na^+^–PC in the first solvation shell (Fig. 6d and e). The enhanced Na^+^–anion interaction in MUL electrolyte reduces the binding affinity between Na^+^ and solvent molecules, enabling more anions (DFOB^−^, DFP^−^, and TFSI^−^) to displace solvent molecules within the solvation sheath. Furthermore, the strong Na^+^–anion interactions effectively restrict the migration of free solvent molecules to the electrode interface, thereby suppressing solvent decomposition and promoting the formation of an inorganic-rich CEI. Additionally, the weakened Na^+^–solvent interaction lowers the desolvation energy barrier for Na^+^, resulting in enhanced reaction kinetics. Pouch cells serve as an ideal platform for comprehensive evaluation of electrolyte performance. In a pouch full cell composed of an HC anode and NFM cathode, the cell employing the MUL electrolyte delivered a reversible capacity exceeding 1550 mA h g^−1^ over 100 cycles (Fig. 6f). In contrast, the cell using the STD electrolyte exhibited severe instability. This instability originates from the pronounced oxidative decomposition of PC in the NFM‖HC system with the STD electrolyte, which impedes stable cell operation. This strategy, by tailoring the interactions among Na^+^, anions, and solvent molecules, reinforces anion–solvent competition within the Na^+^ solvation sheath, offering an innovative pathway to enhance the high-voltage stability of electrolytes for SIBs.

Furthermore, Yu *et al.* developed a low-concentration dual-salt carbonate electrolyte for SMBs. The combination of NaFSI and NaOTF effectively modulates the solvation structure, reduces the desolvation energy barrier, and enhances the low-temperature kinetics of Na^+^ ions.^74^ This dual-salt system promotes the formation of an anion-dominated solvation structure and a robust inorganic-rich SEI layer. Such an innovative electrolyte design significantly improves the ion transport kinetics and extends the cycling lifespan under low-temperature conditions.

Synergistic effects among different sodium salts provide complementary and optimized performance. However, it is crucial to recognize that such complex electrolyte formulations inevitably increase computational demands during simulations. At present, solvation-related investigations primarily involve interactions between cations and anions, yet interactions among anions are also worth consideration, which will be a viable direction for future research.

## 4 Anion-based additives

Electrolyte additives, despite their low dosage, play a crucial role in determining the overall performance of electrolytes and batteries, representing one of the most cost-effective strategies for enhancing battery performance.^75–78^ The incorporation of small amounts of functional additives can selectively improve specific battery characteristics, such as protecting high-voltage cathodes,^79,80^ enabling high energy density,^81,82^ enhancing cycling performance,^83,84^ and improving safety.^85,86^ Reducing the amount of sodium salt and using it as an additive has become a common strategy.^87–89^ In this section, we will categorize recent studies on sodium salt anions used as additives based on the elements contained in the anions. Drawing from the previous discussion on primary salt anions, it is anticipated that anionic additives can lead to a qualitative leap in battery performance.

### 4.1 Fluorine-containing anionic additives

Fluorine-containing additives have been extensively studied due to their ability to form effective passivation layers on both anodes and cathodes.^90,91^ Li *et al.* introduced trifluoroacetate (TFA^−^), an anion with ultra-high electron density, into a diglyme (G2)-based electrolyte as a co-solvent to enhance anion-derived solvation over a wide temperature range.^92^ Owing to its high donor number (34.0 kcal mol^−1^), TFA^−^ exhibits a strong interaction with Na^+^, enabling it to effectively coordinate within the inner solvation shell and reduce the solvent coordination number. This strong cation–anion interaction facilitates the formation of an anion-dominated solvation structure and promotes the desolvation of Na^+^.^21^ The highly coordinated TFA^−^ leads to more PF_6_^−^ anions and fewer G2 molecules occupying the inner solvation shell of Na^+^, favoring the establishment of enhanced Na^+^-anion coordination. This unique solvation configuration induces the formation of an anion-derived, inorganic-rich, and robust SEI layer. As a result, the average mechanical strength of the SEI was significantly enhanced from 0.86 to 5.20 GPa, concurrently with improved stability and reversibility of Na plating/stripping.

Li *et al.* proposed a self-assembled protective layer by applying a perfluorinated anion additive as a dual-chain electrolyte surfactant to enhance the oxidative stability of ether-based electrolytes.^93^ The perfluorinated anion additive, characterized by a lower HOMO–LUMO energy gap, exhibits high reactivity, indicating a greater propensity for decomposition. The preferential adsorption and oxidation of the additive suppress weak oxidation at low voltage, thereby widening the electrochemical window and potentially forming the CEI rich in C–F components and NaF. Furthermore, molecular electrostatic potential analysis revealed that sodium pentadecafluorooctanoate (SPFO) exhibits high electron density centered on the perfluorinated chain and a uniformly distributed charge profile (Fig. 6g), which is expected to interact with the positive electrostatic potential localized on the –C–H groups of DEGDME. When the electron-donating –C–F groups interact with –C–H groups, the fluorine atoms activate the –C–H bonds, leading to weak –C–F⋯H–C– interactions. These weak interactions, defined by Bergström *et al.* as pseudo-hydrogen bonds, enable the SPFO–solvent complex to form a well-compatible structure with homogeneous charge distribution. This is anticipated to suppress electrolyte volatility and enhance thermal stability. The evolution of adsorbed species was monitored using electrochemical *in situ* sum frequency generation (SFG) spectroscopy. Upon applying a 50 mV bias until steady state in two practical systems, the intensities of –CH_2_ (*ν*) at 1430 cm^−1^ and –CH_3_ (*ν*) at 1380 cm^−1^ from DEGDME decreased after the introduction of SPFO (Fig. 6h). This confirms that under an electric field, SPFO preferentially adsorbs at the interface and self-assembles into an ordered layer, effectively shielding the electrode from solvent contact. Moreover, the weak –C–F⋯H–C– pseudo-hydrogen bonds contribute to a significant improvement in the thermal stability of the electrolyte, with an operational tolerance up to 60 °C. In addition, Hagiwara *et al.* used NaDFP as a strategy for optimizing the performance of sodium-metal ion batteries.^94^ As previously discussed, NaDFP exhibits strong cation–anion interaction energy. In this study, the incorporation of 1 wt% NaDFP resulted in high-rate capability and long cycling life. The enhanced electrochemical performance of Na‖HC cells further indicates the beneficial role of NaDFP in facilitating the formation of a stable SEI layer, thereby suppressing overpotential and interfacial resistance.

The core function of fluorine-containing additives is to regulate interfacial chemistry and enhance the mechanical strength of interfacial films. However, an overabundance of inorganic fluorinated components may cause excessive interfacial thickness and increased impedance. Therefore, balancing organic and inorganic components to construct gradient interfacial layers with both high modulus and high toughness, represents the key scientific concern for fluorine-containing anionic additives.

### 4.2 Boron-containing anions as additives

**Fig. 7:**
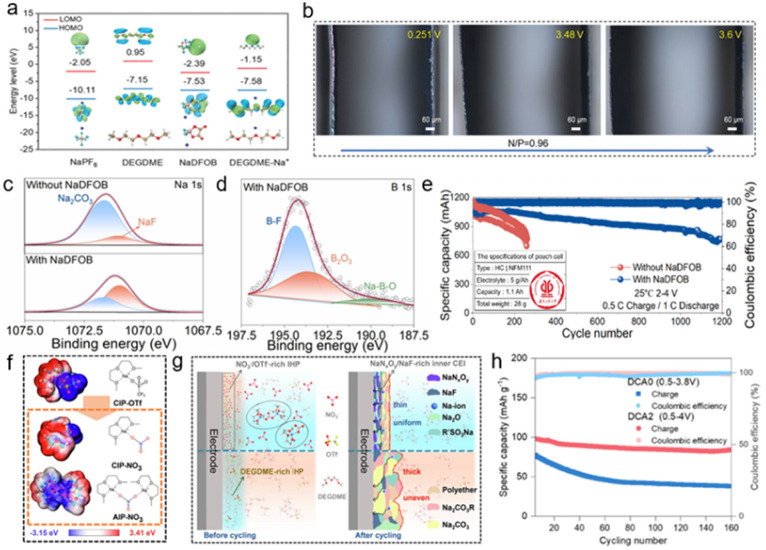
(a) HOMO and LUMO energy levels of NaPF_6_, DEGDME, NaDFOB, and DEGDME–Na^+^ ions. (b) *In situ* optical microscope observations of full cells with a N/P ratio of 0.96 at various voltages during the charging process with the NaDFOB additive.^96^ Copyright 2024 Wiley-VCH GmbH. (c) Comparison of XPS Na 1s spectra of the sodium metal anode in the Na‖Na symmetric cells and (d) B 1s spectra of the sodium metal in the electrolyte with NaDFOB after 10 cycles. (e) Long-term cycling performance of the HC‖NFM111 pouch cell at a charging rate of 0.5C and a discharging rate of 1C.^98^ Copyright 2025 Elsevier. (f) Comparative electrostatic potential mapping and structural formulae of the predominant solvation structures. (g) Schematic illustration of electrolyte environments and CEI transformations induced by the NaNO_3_ additive.^102^ Copyright 2024 Springer Nature. (h) Cycling stability of the NVP‖graphite battery with DCA2 and DCA0 at 0.2C.^103^ Copyright 2025 Wiley-VCH GmbH.

Boron-containing additives represent a highly important and successful category of electrolyte additives. The difluoro(oxalato)borate (DFOB^−^) anion is the most common boron-containing anion, as it exhibits the lowest unoccupied molecular orbital energy level and the highest occupied molecular orbital energy level when coordinating with either Na^+^ or Li^+^. This electronic structure suggests that DFOB^−^ can preferentially undergo reactions at both the cathode and anode, facilitating the formation of a stable electrode–electrolyte interface.^95^ Leveraging this preferential reactivity of DFOB^−^ (Fig. 7a), Qiu *et al.* demonstrated that DFOB^−^ rapidly forms a stable passivation layer on the electrode surface.^96^ A stable SEI rich in NaF and B is formed on the HC surface, which effectively alleviates the excessive reduction and decomposition of electrolyte in the initial charge process and inhibits the repeated dissolution and regeneration of an unstable SEI in the subsequent cycle. On the cathode side, preferential oxidation of NaDFOB enhances the CEI and ensures interfacial stability of the FeMn-based Prussian blue cathode. Furthermore, the pre-formed CEI, composed of sturdy NaF and boron-rich components, prevents undesirable oxidative decomposition of the electrolyte and dissolution of transition metals. The formed uniform and mechanically robust SEI and CEI collaboratively suppress the formation of detrimental sodium dendrites. Even at a low N/P ratio of 0.96, no dendrites were observed throughout the entire charging process (Fig. 7b).

Similarly, Xia *et al.* combined theoretical calculations and experimental evidence to show that the DFOB^−^ anion exhibits a lower adsorption energy than other electrolyte components, leading to its preferential adsorption within the inner Helmholtz plane near both electrode surfaces. Introducing a small amount (0.5 wt%) of NaDFOB—a borate additive with electron-rich characteristics—into the electrolyte system enables the construction of a robust inorganic-rich EEI *via* an anion-preferential adsorption and decomposition mechanism, thereby effectively suppressing side reactions.^97^ As a result, the Na_*x*_Ni_0.25_Fe_0.25_Mn_0.5_O_2_ cathode, HC anode, and the corresponding full cell achieve outstanding cycling performance. Liu *et al.* used NaDFOB as an additive in the Na^+^ solvation sheath in SMBs.^98^ This promotes anion aggregation *via* van der Waals interactions, leading to the formation of the SEI enriched with NaF and B_2_O_3_ (Fig. 7c and d), which significantly enhances the stability of both the SEI and CEI. This modification has been shown to effectively suppress transition metal leaching from the cathode and sodium dendrite growth on the anode. Further evaluation using pouch cells confirms the potential commercial applicability of NaDFOB as a functional electrolyte additive (Fig. 7e).

Beyond its established role in lithium batteries, LiDFOB has found key applications in SIBs. Chen *et al.* explored its function as an electrolyte additive for improving SIB cathode performance.^99^ They demonstrated that LiDFOB effectively inhibits electrolyte decomposition and HF-induced corrosion at the electrode surface. By contributing to the formation of a more robust CEI, it shields the active material from damage, thereby inhibiting the dissolution of transition metals.

In addition to the classic boron-containing anion DFOB^−^, BF_4_^−^ is also a highly cost-effective boron-containing anion. Even with minimal addition, it can significantly enhance the performance of the entire battery system at a very low cost. Yang *et al.* introduced a trace amount of NaBF_4_ into a G2-based electrolyte, enabling stable cycling of the Na_3_(VOPO_4_)_2_F cathode.^100^ This work provides valuable insights into the application of ultra-low concentration ether-based electrolytes in durable high-voltage SIBs. The improved cycling stability can be attributed to the modulation of the electrolyte solvation structure by NaBF_4_. Specifically, BF_4_^−^ anions weaken the interaction between G2 molecules and Na^+^, while introducing more PF_6_^−^ into the Na^+^ solvation sheath. This promotes the enrichment of inorganic species within the CEI, mitigates its dissolution under high voltage, and facilitates stable long-term cycling. Similarly, Yan *et al.* applied ether-based electrolyte containing NaBF_4_ as the main salt or additive in the NFM‖HC full cell, which exhibited superior performance compared to an electrolyte with NaPF_6_.^101^ This improvement is attributed to the stronger interaction between BF_4_^−^ and Na^+^, which reduces the solvation of Na^+^ with solvent molecules, thereby promoting the formation of a CEI and SEI rich in inorganic species. Furthermore, investigations *via* SEM, XPS, EDS, and solubility tests revealed that BF_4_^−^ facilitates the formation of a more stable EEI enriched with boron-containing and inorganic compounds.

Unlike previous fluorinated anions, boron-based anions offer a more pronounced contribution to the cathode CEI. Simultaneously, when the empty orbital remains uncoordinated, the central boron atom acts as a Lewis acid to scavenge trace moisture and HF. Designing boron-containing anions requires integrating organic and inorganic groups, such as coupling boron with fluorinated functional groups to enable simultaneous action at both electrode interfaces.

### 4.3 Nitrogen-containing anions as additives

Nitrogen-containing additives, such as amines, nitriles, and nitrates, have been widely employed in electrolytes. To address the challenge of ether-based electrolyte oxidation under high voltage, Xia *et al.* introduced NO_3_^−^ anions to passivate the reactive terminal hydrogen sites of diglyme molecules.^102^ The incorporation of this anionic additive induces rearrangement of the primary solvation structure. MD simulations reveal that NO_3_^−^ ions are incorporated into the inner solvation sheath, and their electron-donating ability weakens Na^+^–solvent interactions, thereby enhancing ionic conductivity. DFT calculations further demonstrate that the electron-donating NO_3_^−^ shifts the solvation structures toward the CIP and even promotes the formation of aggregates (Fig. 7f). Moreover, sodium nitrate plays a dual role in the chemistry of CEI formation. The inclusion of NO_3_^−^ anions in the primary solvation shell releases some OTF^−^ anions into the free ion state. These anions accumulate in the IHP, where they decompose to form a CEI rich in NaF and RSO_3_Na/RSO_2_Na species, which act as effective electron insulators and suppress further solvent oxidation (Fig. 7g). Meanwhile, the preferential decomposition of NO_3_^−^ ion serves as a sacrificial process, incorporating NaN_*x*_O_*y*_ compounds into the CEI and protecting the C–O bonds in G2 molecules from oxidation. This synergy results in a thin, dense CEI layer that selectively permits Na^+^ transport while blocking electron transfer.

Recently, Wu *et al.* proposed the concept of bridging-donor-ligands, which proved that the NaDCA additive used the dicyandiamide anion (DCA^−^) as a bridging ligand of the graphite layer, promoting the formation of rich ligand channels and significantly enhancing the structural stability of ternary graphite intercalation compounds.^103^ During the initial discharge process, DCA^−^ acts as a ligand and driven by its strong electron-withdrawing nature, it actively intercalates into the graphite interlayers. Upon charging, DCA^−^ serves as an electron donor continuously supporting the graphite layers under the influence of the electric field, thereby mitigating anode volume fluctuations during cycling. In NVP‖graphite full cells, this mechanism enables a capacity retention of approximately 80% after 160 cycles (over 1280 hours) (Fig. 7h). This mechanism reveals new potential for achieving high-energy-density graphite anodes in SIBs.

The value of nitrogen-containing anions derives from their unique decomposition products, such as Na_3_N with high ionic conductivity in the interfacial films. The challenges are the limited solubility of some salts and precise control of their decomposition pathways.

## 5 Conclusion and outlook

This review systematically summarizes and elaborates on the latest advancements in research concerning anion-based electrolytes for SIBs, focusing primarily on the critical mechanisms by which solvation structures induced by single or composite anions optimize battery electrochemical performance, enhance interfacial ion transport kinetics, and improve EEI stability. It specifically elucidates how diverse types of anions, leveraging their distinct coordination capabilities and modes, effectively regulate the solvation environment of Na^+^. This regulation facilitates efficient and rapid ion migration at the interface, significantly reduces the desolvation energy barrier for Na^+^ at the interface, and promotes the formation of denser, more stable interfacial protective layers—particularly the inorganic-rich SEI and CEI. Notably, introducing specific anions as functional additives into the electrolyte system not only directly participates in and optimizes the primary solvation sheath structure of Na^+^ but also induces the formation of stable, ion-conductive interfacial phases at the electrode surfaces, thereby holistically enhancing the battery's cycle life and safety. Furthermore, this paper critically reviews the remarkable efficacy demonstrated by recently developed novel anions and their corresponding sodium salts in advancing the performance of SIBs.

Based on a systematic analysis of recent significant research efforts, constructing anion-centered solvation structures has transcended the conventional strategy employed in HCEs, which solely relies on increasing the anion-to-solvent ratio. Several innovative and highly efficient regulatory pathways have emerged: (i) modulating the Na^+^ coordination environment *via* weakly coordinating solvents—for instance, utilizing solvents with inherently weak coordination capabilities such as fluorinated ethers or linear ethers effectively weakens the interactions between solvent molecules and Na^+^. This creates favorable conditions for anions to enter the inner solvation sheath, thereby enhancing their participation within the coordination structure; (ii) employing strongly associating sodium salts to boost anion coordination competitiveness—sodium salts exhibiting high Na^+^ binding energy, including NaBF_4_, NaOTF, and NaTFA, substantially elevate the advantage of anions in the coordination competition. This facilitates their easier entry and stable accommodation within the primary solvation shell of Na^+^, resulting in anion-enriched coordination architectures. Table 1 summarizes the cation–anion binding energies for typical sodium salts; (iii) introducing functional additives or leveraging solvent synergistic effects—specific additives enable precise tailoring of the composition and configuration of anion-containing solvation sheaths. Meanwhile, synergistic interactions among diverse solvent molecules contribute to forming more loosely packed and flexible solvation layers, promoting greater anion involvement in coordination. These approaches collectively optimize interfacial ion transport and enhance interfacial stability. Furthermore, the distinct effects of anion coordination ability on electrolyte ionic conductivity and viscosity must be comprehensively considered. While strongly coordinating anions can reside more stably within the sodium ion's solvation sheath and effectively participate in interfacial film formation, they often lead to increased viscosity and reduced salt solubility. To mitigate these negative effects stemming from strong coordination, several effective strategies have been developed: one approach involves introducing diluents to construct localized high-concentration electrolytes. This strategy can significantly enhance anion involvement in the solvation structure and interfacial reactions while maintaining low overall viscosity. Another line of strategy focuses on designing weakly coordinating anions (*e.g.*, TFSI^−^and FSI^−^) from the outset or utilizing the entropy effect of solvent mixing to induce the formation of weakly solvating structures. These methods have proven effective in markedly improving the ionic conductivity of the system.

**Table 1:** Binding energy and anion ball-and-stick model of typical sodium salts

Salt	Anion structure	Binding energy (eV)
NaPF_6_	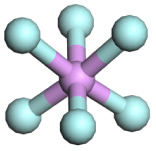	−5.08
NaClO_4_	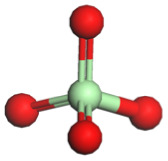	−5.23
NaBF_4_	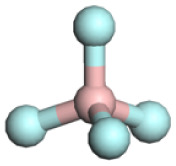	−5.55
NaFSI	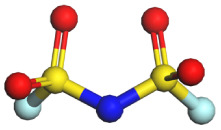	−4.85
NaTFSI	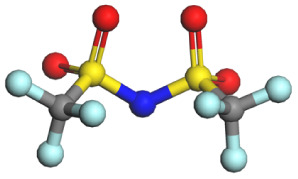	−4.98
NaOTF	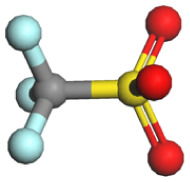	−5.24
NaDFOB	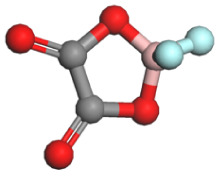	−5.32
NaTFA	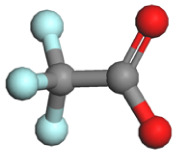	−5.73
NaNO_3_	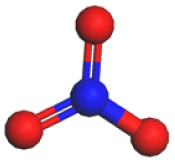	−5.68

In summary, by precisely regulating the solvation behavior of anions and their interfacial chemical reactions, anion-directed electrolyte design strategies play a pivotal role in holistically enhancing the comprehensive electrochemical performance, interfacial compatibility, and long-term cycling stability of SIBs. Building upon current research achievements and remaining challenges, future efforts should focus on exploring novel high-performance anionic sodium salts, deepening studies on multi-anion synergistic mechanisms, developing anion-modulation strategies tailored for high-voltage cathodes/high-capacity anodes, and advancing the integration, validation, and application of relevant electrolyte systems within practical battery configurations. These endeavors will drive research toward higher-performing, more practical anion-dominated SIB electrolytes.

Anion-regulated electrolytes demonstrate pivotal roles in enhancing the overall electrochemical performance, interfacial compatibility, and long-term cycling stability of SIBs by modulating the behavior of anions within solvation structures and their interfacial chemical processes. Building upon current research progress and existing challenges, we systematically propose the following future research directions to drive sustained advancement in this field.

### 5.1 Sustained innovation in electrolyte systems with systematic optimization of core components

Innovation in electrolyte formulations represents a critical pathway toward high-performance SIBs. Future efforts should prioritize the systematic optimization of three core constituents: sodium salts, solvents, and additives. For sodium salts, emphasis should be placed on developing functional sodium salts featuring novel anion structures to enhance anion participation in solvation architectures, thereby establishing more stable and efficient anion-dominated solvation sheaths. Furthermore, exploration of single-salt and multi-salt synergistic systems is essential to harness collaborative effects among diverse anions for precise control over solvation behavior. In view of the abundance of sodium relative to lithium, the commercial scale production of new sodium salts needs to focus on optimizing the synthesis routes and improving atomic economy. Solvent design requires identifying or engineering environments that promote effective anion coordination, fostering the formation of inorganic-rich, stable interfacial layers (CEI/SEI) to improve safety and cycling lifespan. Additives play a decisive regulatory role; molecules bearing specific functional groups can fine-tune anion behavior within solvation sheaths, reinforcing their dominance and leveraging anion-derived interfaces to enhance interfacial compatibility, thermal stability, and chemical robustness.

### 5.2 Deepening the guiding role of theoretical calculations and simulations in electrolyte research

Theoretical modeling and simulation offer irreplaceable insights into structure–property relationships and underlying mechanisms. DFT is widely applied to evaluate redox stability—for example, by calculating HOMO/LUMO energy levels of solvent molecules to assess electrochemical windows. DFT further enables analysis of Na^+^–anion binding energies, coordination competition dynamics, and charge distribution patterns, providing a foundational understanding of solvation structures. MD simulations complement this by capturing dynamic evolution of solvation configurations, ion transport kinetics, physicochemical property transitions, and interfacial reaction mechanisms at the molecular level. Integration of multiscale theoretical tools (*e.g.*, DFT + MD) facilitates quantitative dissection of solvation motifs, unraveling complex interactions among anions, cations, and solvent molecules. These approaches not only simulate key processes but also guide rational electrolyte design and predict electrochemical behavior in practical systems.

### 5.3 Accelerating material discovery *via* machine learning in electrolyte development

Machine learning (ML), as a data-driven accelerator, is increasingly vital for expediting novel electrolyte discovery, predicting critical performance metrics, and optimizing battery systems.^104^ A central challenge in anion-regulated electrolytes is achieving optimal anion–solvent pairing. ML addresses this through structure–activity relationship models that predict electrochemical performances of emerging anions or solvation configurations, enabling rapid screening of candidate systems. Strategies include high-throughput virtual screening based on physicochemical descriptors and electrochemical databases, allowing evaluation of thousands of anion/solvent/additive combinations. ML integrates multi-source data (experimental + computational) to comprehensively understand electrolyte behavior. Synergy between theoretical calculations and ML will drive rational design and innovation in anion-centric electrolytes, supporting sustainable advancement of sodium battery technology.

### 5.4 Advancing advanced characterization methods to decode solvation structures and interfacial evolution

Solvation structures and their derived interphases are central to optimizing anion-regulated electrolytes. While theoretical simulations provide insights, experimental characterization remains indispensable for directly probing these phenomena. Spectroscopic techniques (*e.g.*, Raman spectroscopy) currently analyze bulk solvation structures but lack spatial/temporal resolution for microscopic details—such as ion rearrangement at electrode/electrolyte interfaces, local coordination evolution, or dynamic solvation sheath changes during cycling.^105^ To bridge this gap, cutting-edge *in situ*/*operando* characterization tools with high spatiotemporal resolution are urgently needed to monitor real-time interfacial formation, structural evolution, and property shifts under operational conditions. Microscopy (SEM/TEM) offers valuable insights into interfacial morphology and composition. Multimodal platforms combining spectroscopy, spectrometry, and imaging will elucidate correlations between solvation structures and interfacial evolution, advancing toward precision-driven, dynamic, and mechanism-guided design of anion-regulated electrolytes.^106,107^

Looking ahead, research on anion-regulated electrolytes will increasingly feature deep multidisciplinary convergence. By establishing a closed-loop “design–simulation–validation” paradigm integrating theoretical calculations, machine learning, and advanced characterization techniques, precise decoding and proactive control of solvation structures and interfacial processes will be progressively realized. This approach will systematically advance electrolyte systems from single-component optimization toward multi-component synergistic design, shifting focus from macroscopic performance enhancement to microscopic mechanism-driven innovation. With deepened understanding of anion behavior, it is anticipated that next-generation electrolyte systems combining high ionic conductivity, wide electrochemical windows, and self-healing interfacial functionality will emerge. Such advancements will not only significantly elevate the energy density, cycle life, and safety performance of SIBs but also offer innovative electrolyte design paradigms for the broader alkali metal battery family. Ultimately, through multiscale, end-to-end collaborative innovation, anion-regulation strategies hold promise to break through current performance bottlenecks in SIB technology, providing critical material foundations for low-cost, high-efficiency, and high-safety sustainable energy storage systems—thereby playing a vital supporting role in global energy transition.

## Author contributions

S. Li: investigation, writing – original draft, and writing – review & editing; Y. Heng: investigation, writing – original draft and format analysis; Z. Gu: supervision and writing – review & editing; X. Wang: investigation and conceptualization; Y. Liu: formal analysis; X. Zhang: modification; Z. Sun: writing – review & editing; D. Liu: formal analysis; B. Li: formal analysis; X. Wu: resources, writing – review & editing, and supervision.

## Conflicts of interest

There are no conflicts to declare.

## Data Availability

No primary research results, software or code have been included and no new data were generated or analysed as part of this review.
